# Data from docking simulations to develop an efficient strategy able to evaluate the interactions between RAGE and MDA-induced albumin adducts

**DOI:** 10.1016/j.dib.2017.05.009

**Published:** 2017-05-06

**Authors:** Angelica Mazzolari, Crescenzo Coppa, Alessandra Altomare, Genny Degani, Giulio Vistoli

**Affiliations:** a Dept. Pharmaceutical Sciences, University of Milan, Via Mangiagalli, 25, I-20133 Milan, Italy; b Department of Biosciences, University of Milan, Via Celoria 26, I-20133 Milan, Italy

## Abstract

This data article contains the results of docking simulations performed in order to develop a suitable in silico strategy able to assess the stability of the putative complexes between RAGE and MDA induced adducts on human albumin as experimentally determined doi: 10.1016/j.redox.2016.12.017, (Degani et al., 2017) [1]. The docking simulations involved different approaches to give a simplified yet realistic representation of the protein adducts and their environment. With increasing complexity, simulations involved the corresponding albumin tripeptides and pentapeptides with the modified residue in the central position as well as pseudo-structures which were generated by collecting the albumin residues around the adducted residue within a sphere of 7.5 Å and 5 Å radius. The reliability of the tested approaches was assessed by monitoring the score differences between adducted and unmodified residues. The obtained results revealed the greater predictive power of the spherical pseudo-structures compared to the simple tri- or pentapeptidic sequences thus suggesting that RAGE recognition involves residues which are spatially close to the modified residue even though not necessarily adjacent in the primary sequence.

**Specifications Table**

**Table:** 

Subject area	*Biology*
More specific subject area	*Molecular modeling studies of protein-protein complexes*
Type of data	*Data extracted from docking simulations and represented by putative complexes and docking scores within the text*
How data was acquired	*Molecular docking simulations using PLANTS*
Data format	*Binding features and structural parameters graphically analyzed*
Experimental factors	*NMR resolved structure for RAGE, X-ray resolved structure for human albumin, MDA-based adducted residues as identified by MS.*
Experimental features	*Docking simulations using ChemPLP as score function.*
Data source location	*Milan, Italy*
Data accessibility	*The coordinates of complexes depicted in*Fig. 1*are included as PDB files*

**Value of the data**

• A comparison of the tested approaches reveals the superior capacity of surrounding spheres made of exposed residues to represent the environment of the adducted residues
• In contrast, sequence-based environments yield worse docking results and are unable to account for the adduct accessibility.
• These results suggest that RAGE recognition is not limited to residues adjacent in sequence but involves all residues spatially close to the adduct.
• The proposed approach can be conveniently applied to other protein–protein docking simulations in which attention is focused on well-defined regions
• Even though the used docking scores are usually utilized to parameterize the interactions of small ligands they proved successful in describing protein–protein interactions.

## 1 Data

The data were generated by four sets of docking simulations involving the RAGE structure and simplified structures representative of increasing complexity for the MDA induced albumin adducts with a view to evaluating the corresponding protein–protein interactions as experimentally determined [1]. In detail, the four sets concerned: (1) the albumin tripeptides with the modified residue in the middle; (2) the albumin pentapeptides with the modified residue in the middle; protein pseudo-structures as generated by collecting the accessible residues around the modified residue (3) within a 5 Å radius sphere and (4) within a 7.5 Å radius sphere. For all tested approaches, docking simulations involved the so generated representative protein ligands with and without the detected adductions.

**Fig. 1:**
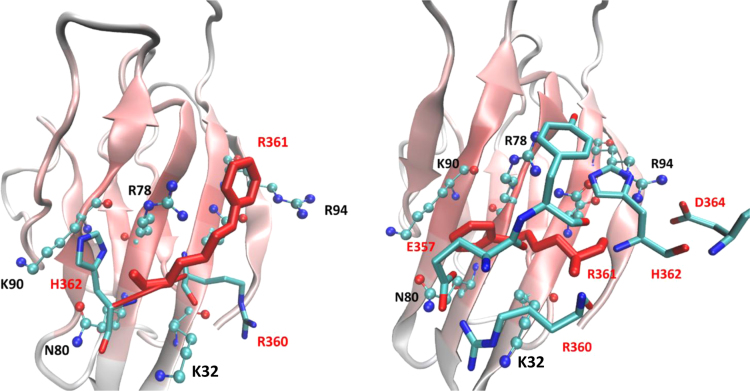
Putative complexes as generated for the Arg361 adducted residue by using the tripeptide (1A) and the surrounding 5 Å radius sphere (1B). One may note that the destabilizing effects as seen in the tripeptide complex are mostly due to the ionic repulsion between Arg362 and Lys32 (of RAGE) and constrain the adducted residue in a superficial pose; in contrast, the inclusion of surrounding 5 Å radius sphere allows a more extended set of beneficial interactions to be considered (such as Asp364 and Arg92) thus reducing the repulsive effect exerted by Arg362 and allowing a tighter arrangement of the adduct.

Table 1 compares, for a set of representative docking functions, the average values as computed for the modified and unmodified protein ligands. For the APBS score which encodes for ionic contacts, Table 2 compares the scores for each adducted and unmodified residue as computed by the four tested strategies. For the residue showing the largest score differences between the tested computational procedures (i.e. Arg361), Fig. 1A and B compares the corresponding complexes for the adducted residue as generated by using the corresponding tripeptide and the surrounding 5 Å radius sphere.

**Table 1:** Score averages and relative differences (in bold, *Δ*=Score_adducted_−Score_unmodified_) of adducted and unmodified albumin structures. The table includes five representative scores among which two are general functions (ChemPLP and XScore) and three encode for specific interaction types such as hydrophobic (MLPInS), Van der Waals (CHARMM) and ionic (APBS).

*Ligands*	*Adducts*	*MLPInS*	*CHARMM*	*APBS*	*CHEMPLP*	*XScore*
*Sphere 7.5 *Å	*DHPLys+RP*	**−***57.68*	**−***68.59*	**−***47.41*	**−***22.86*	**−***5.85*
*Sphere 7.5 *Å	*No*	**−***86.48*	**−***55.19*	**−***50.99*	**−***40.91*	**−***5.70*
***Sphere 7.5 *****Å**	***Δ***	***28.80***	**−*****13.40***	***+3.58***	***+18.06***	**−*****0.15***
*Sphere 5.0 *Å	*DHPLys+RP*	**−***54.11*	**−***51.77*	**−***53.74*	**−***65.42*	**−***7.18*
*Sphere 5.0 *Å	*No*	**−***61.82*	**−***33.87*	**−***36.30*	**−***47.64*	**−***5.47*
***Sphere 5.0 *****Å**	***Δ***	***+7.71***	**−*****17.90***	**−*****17.44***	**−*****17.78***	**−*****1.71***
*Tripeptides*	*DHPLys+RP*	**−***11.42*	**−***32.13*	**−***8.10*	**−***58.82*	**−***4.65*
*Tripeptides*	*No*	**−***40.74*	**−***21.93*	**−***21.93*	**−***43.52*	**−***4.31*
***Tripeptides***	***Δ***	***+29.31***	**−*****10.20***	***+13.83***	**−*****15.30***	**−*****0.34***
*Pentapeptides*	*DHPLys+RP*	**−***23.06*	**−***27.95*	**−***30.88*	**−***56.36*	**−***4.46*
*Pentapeptides*	*No*	**−***44.88*	**−***21.90*	**−***19.01*	**−***51.76*	**−***4.24*
***Pentapeptides***	***Δ***	***+21.82***	**−*****6.05***	**−*****11.87***	**−*****4.59***	**−*****0.23***

All score averages are reported in kcal/mol apart from APBS which is in kJ/mol and MLPInS which is dimensionless.

**Table 2:** Specific APBS scores (and relative difference in bold, *Δ*=Score_adducted_**−**Score_unmodified_) for the six adducted residues considered by all approaches. Even though the lack of experimental data concerning the specific role of single albumin adduct in RAGE binding does not allow a detailed rationalization, these scores confirm the greatest reliability of the approach based on 5 Å sphere and suggest that Lys75, Lys337 and Arg361 should play key roles in RAGE recognition. Even though the present study was designed to assess which computational procedure performs best, the tested approaches can be also exploited in a sort of consensus strategy by verifying which residues show coherent score values. In this respect, Lys75 and Arg361 are the only residues exhibiting negative score differences (i.e. adducted residue which interacts stronger than the unmodified one) in all four cases.

*residues*	*Adduct*	*APBS Tripeptides*	*APBS Pentapeptides*	*APBS 5A sphere*	*APBS 7.5A Sphere*
k75	*DHPLys*	1.37	2.97	**−**64.30	**−**63.60
k75	*No*	5.18	9.10	**−**34.90	**−**23.95
**k75**	***Δ***	**−3.81**	**−6.13**	**−29.40**	**−39.65**
k337	*DHPLys*	**−**20.20	**−**44.50	**−**95.00	**−**12.20
k337	*No*	**−**21.60	**−**46.60	**−**58.98	**−**5.32
**k337**	***Δ***	**+1.40**	**+2.10**	**−36.02**	**−6.88**
k499	*DHPLys*	**−**1.97	0.99	**−**33.30	**−**69.80
k499	*No*	**−**3.93	**−**7.94	**−**37.63	**−**69.76
**k499**	***Δ***	**+1.96**	**+8.93**	**+4.33**	**−0.04**
k588	*DHPLys*	**−**24.00	**−**93.40	**−**97.60	**−**48.70
k588	*No*	**−**40.90	**−**57.10	**−**80.89	**−**103.39
**k588**	***Δ***	**+16.90**	**−36.30**	**−16.71**	**+54.69**
k598	*DHPLys*	**−**0.45	10.40	**−**72.20	**−**96.30
k598	*No*	4.18	6.81	**−**42.50	**−**39.95
**k598**	***Δ***	**−4.63**	**+3.59**	**−29.70**	**−56.35**
r361	*RP*	9.98	11.40	**−**52.30	**−**7.55
r361	*No*	17.00	18.50	**−**4.12	**−**6.88
**r361**	***Δ***	**−7.02**	**−7.10**	**−48.18**	**−0.67**

## 2 Experimental design, materials and methods

### 2.1 Preparation of the RAGE structure and of the representative adducted and unmodified albumin ligands

The RAGE structure and the initial albumin structure were selected and prepared as described in the reference paper [1]. Briefly, the resolved recombinant structure of HSA was selected (PDB ID: 4G03) and utilized to generate the found MDA-induced adducts by manually modifying the arginine and lysine residues as listed in Table 2. Again the resolved structure of the RAGE V-domain in complex with a hydroimidazolone adduct (PDB ID: 2mov) was chosen since it allows a precise definition of the binding site to be used in the following docking simulations. As mentioned above, docking simulations involved four different strategies in order to generate simplified yet realistic albumin ligands which were able to represent the protein environment which surrounds the adducted residues and which is reasonably involved in RAGE recognition. Aimed at analyzing which approach affords, on average, the largest score difference between adducted and unmodified albumin structures, these different approaches also have the objective of elucidating how much the environment surrounding the adducted residue influences the RAGE binding. With regard to albumin set-up, the adducted residues as detected by MS analyses, were manually modified using the entire albumin structure which was then minimized to preserve the experimental folding while allowing a satisfactory arrangement of the inserted adducts. The so adducted and optimized albumin structure, along with the unmodified structure, was utilized in the following docking simulations.

The first two docking strategies involved the tripeptides and the pentapeptides with the modified residue in the middle as extracted from albumin structure. In this way, the following tripeptides were simulated: V64K65L66, A74K75T76, D280R281A282, S297K298L299, S336K337D338, R360R361H362, K437K438V439, T498K499C500, D587K588E589 and K597K598L599. Similarly, the following pentapeptides were considered: H63V64K65L66V67, P73A74K75T76C77, D279D280R281A282D283, S296S297-K298L299K300, E335S336K337D338V339, A359R360R361H362P363, T436K437K438V439P440, V497T498K499C500C501, D597D587K588E589T590 and G596K597K598L599V600. In detail, the simulated tri- and pentapeptides were extracted from the albumin (modified and unmodified) structures and then completed by transforming the two termini in amide functions. The obtained peptides were not further optimized to preserve the original folding.

The other two docking campaigns were focused on the protein environments of the adducted residues as obtained by generating pseudo-structures composed of the adducted residue plus the neighboring accessible residues comprised within a 5 Å or 7.5 Å radius sphere. While the previous sequence-based approaches involved all adducted residues regardless of their exposure, these environment-based simulations were focused only on the residues which possess a satisfactory degree of accessibility as assessed by a residue surface greater than 30.0 Å^2^. By using this criterion, these simulations were not applied to Lys65, Lys298, Lys499 and Arg281. In detail, the surrounding residues were collected by using the selection features as implemented in the VEGA suite of programs [2] and again residues with a surface less than 30.0 Å^2^ were deleted. The so selected neighboring and exposed residues were completed when necessary by transforming the cut termini into amide functions and were then merged into a single molecular entity to allow their following simulation by the docking program (see below). On average, the generated spheres include 12 residues with radius equal to 5 Å and 22 residues with radius equal to 7.5 Å. The docking simulations were repeated by considering the same surrounding residues with and without the adduction.

### 2.2 Docking simulations

Docking simulations involved the RAGE structure and the four sets of modified and unmodified albumin peptides as described above. Docking simulations were performed by using PLANTS and focusing the search on a 12 Å radius sphere around the bound hydroimidazolone [3]. For each albumin structure, 10 poses were generated and ranked by ChemPLP score which combines piecewise linear potential (PLP) with terms of GOLD׳s Chemscore. The so obtained best pose was then optimized by using Namd with the CHARMM force field and Gasteiger׳s atomic charges [4] and utilized for rescoring analyses. Rescoring was carried out using the Rescore+ tool [5] as implemented in VEGA and allowed the calculation of an extended set of score functions including overall scores (such as ChemPLP or XScore) plus scores focused on specific interaction types such as APBS for ionic interactions, CHARMM for van der Waals contacts as parameterized by the Lennard-Jones component of the CHARMM force-field and MLP_InS_ (Molecular Lipophilic Potential Interaction Score) for hydrophobic interactions.

## References

[1] Degani G., Altomare A.A., Colzani M., Martino C., Mazzolari A., Fritz G., Vistoli G., Popolo L., Aldini G. (2017). A capture method based on the VC1 domain reveals new binding properties of the human receptor for advanced glycation end products (RAGE). Redox Biol..

[2] Pedretti A., Villa L., Vistoli G. (2002). VEGA: a versatile program to convert, handle and visualize molecular structure on Windows-based PCs. J. Mol. Graph Model.

[3] Korb O., Stützle T., Exner T.E. (2009). Empirical scoring functions for advanced protein-ligand docking with PLANTS. J. Chem. Inf. Model.

[4] Phillips J.C., Braun R., Wang W., Gumbart J., Tajkhorshid E., Villa E., Chipot C., Skeel R.D., Kalé L., Schulten K. (2005). Scalable molecular dynamics with NAMD. J. Comput. Chem..

[5] Pedretti A., Granito C., Mazzolari A., Vistoli G. (2016). Structural effects of some relevant missense mutations on the MECP2-DNA binding: a MD study analyzed by rescore+, a versatile rescoring tool of the VEGA ZZ program. Mol. Inform..

